# A before-after study of hospital use in two frail populations receiving different home-based services over the same time in Vancouver, Canada

**DOI:** 10.1186/s12913-018-3040-y

**Published:** 2018-04-05

**Authors:** Margaret J. McGregor, Michelle B. Cox, Jay M. Slater, Jeff Poss, Kimberlyn M. McGrail, Lisa A. Ronald, John Sloan, Michael Schulzer

**Affiliations:** 1 Department of Family Practice, University of British Columbia, 713-828 West 10th Avenue, Vancouver, BC V5Z 1M9 Canada; 2 Community Geriatric Programs, VCH, Vancouver, Canada; 3 School of Public Health and Health Systems, University of Waterloo, Waterloo, Canada; 4 UBC Centre for Health Services and Policy Research, Vancouver, Canada; 5 UBC School of Population and Public Health, Vancouver, Canada; 6 Pacific Parkinson’s Research Centre, Vancouver, Canada; 7 Vancouver Coastal Health’s Research Institute’s Centre for Epidemiology and Evaluation, Vancouver, Canada

**Keywords:** Community medicine, Family practice/general practice/primary care, Geriatric medicine/care of the elderly, Homecare

## Abstract

**Background:**

As individuals age, they are more likely to experience increasing frailty and more frequent use of hospital services. First, we explored whether initiating home-based primary care in a frail homebound cohort, influenced hospital use. Second, we explored whether initiating regular home care support for personal care with usual primary care, in a second somewhat less frail cohort, influenced hospital use.

**Methods:**

This was a before-after retrospective cohort study of two frail populations in Vancouver, Canada using administrative data to assess the influence of two different services started in two different cohorts over the same time period. The participants were 246 recipients of integrated home-based primary care and 492 recipients of home care followed between July 1st, 2008 and June 30th, 2013 before and after starting their respective services. Individuals in each group were linked to their hospital emergency department visit and discharge abstract records. The main outcome measures were mean emergency department visit and hospital admission rates per 1000 patient days for 21 months before versus the period after receipt of services, and the adjusted incidence rate ratios (IRRs) on these outcomes post receipt of service.

**Results:**

Before versus after starting integrated home-based primary care, emergency department visit rates per 1000 patient days (95% confidence intervals) were 4.1 (3.8, 4.4) versus 3.7 (3.3, 4.1), and hospital admissions rates were 2.3 (2.1, 2.5) versus 2.2 (1.9, 2.5). Before versus after starting home care, emergency department visit rates per 1000 patient days (95% confidence intervals) were 3.0 (2.8, 3.2) versus 4.0 (3.7, 4.3) visits and hospital admissions rates were 1.3 (1.2, 1.4) versus 1.9 (1.7, 2.1). Home-based primary care IRRs were 0.91 (0.72, 1.15) and 0.99 (0.76, 1.27) and home care IRRs were 1.34 (1.15, 1.56**)** and 1.46 (1.22, 1.74) for emergency department visits and hospital admissions respectively.

**Conclusions:**

After enrollment in integrated home-based primary care, emergency department visit and hospital admission rates stabilized. After starting home care with usual primary care, emergency department visit and hospital admission rates continued to rise.

## Background

Frailty is a “multidimensional syndrome of loss of reserves (energy, physical ability, cognition, health) that gives rise to vulnerability” (p. 489 [1]) and is highly associated with older age [2]. It is estimated 5.6% of the general population is homebound as a result of frailty [3] and primary care medical health services are poorly designed for these individuals. The standard office visit lasts 10-15 min, examination tables are often inaccessible to those with mobility problems, and many frail individuals are dependent on others to get to appointments. In addition, the number of family physicians making house calls is declining over time [4].

Frail community-dwelling older people are at greater risk of hospital emergency department (ED) visits and hospital admissions [5, 6]. One Canadian study reported the 2004/5 prevalence of ED visits was 41.7% among those over 85 years [7]. Ironically, while frail older people are most likely to use acute hospital care (ED visits and hospital admissions), they are least likely to benefit [8, 9] and most likely to experience harm from such services [10]. Research has further demonstrated an inverse association between ED use and access to primary care among older adults [11, 12].

Home-based primary care (HBPC) refers to integrated home-based care for medical, nursing, rehabilitative, and palliative care needs. A number of home-based primary care programs have developed throughout North America [13–15]. However, such programs are still uncommon, there are relatively few evaluative studies on these programs, and generalizability is limited by the variation in types of services offered [14], policy context, and the jurisdiction in which they are delivered [13, 16–18].

Home care (HC) is defined as clinical case management, direct nursing care, allied health support and home support as needed [19] with receipt of usual primary medical care as a separate service. For the purposes of this study, HC refers to the need and receipt of regular home support services. There is growing awareness in Canada of the importance of HC as a strategy for strengthening the health system’s capacity to meet the needs of the aging population [20, 21] and HC has become a major focus of recent federal government funding [22, 23]. A major challenge of HC has been its’ separate evolution from primary medical care such that clinical records of HC providers and family physicians remain separate, and communication between these services is often episodic and crisis driven [24].

This study’s first objective was to assess the influence of a multidisciplinary HBPC program in Vancouver, Canada on acute hospital use by examining rates of ED visits and hospital admissions before and after receipt of this service. The second objective was to examine these outcomes in a selection of community-dwelling individuals who started HC over the same time period, and continued to receive usual primary care.

## Methods

### Setting and study populations

#### Home-based primary care (HBPC) cohort

Administered out of a large tertiary care hospital (Vancouver General Hospital), the HBPC program (Home Visits to Vancouver’s Elders – Home ViVE), was started in 2008 to provide primary care to Vancouver’s seniors unable to access usual ambulatory care due to dementia and/or physical frailty. The program includes family physicians and nurse practitioners, each of whom provide longitudinal primary care to a regular panel of patients. The primary care providers are supported by registered nurses, allied health providers, and office administrative support.

HBPC services include planned regular home visits, responsive day-time and after-hours care for emergencies, and nursing, physical and occupational rehab services as needed. The team holds regular meetings to discuss both individual patients and service quality more generally. In addition, there is capacity for allied health providers to communicate with physicians and nurse practitioners through a shared electronic medical record (EMR).

At the time of the study, admission criteria were 65+ years of age and inability to access ambulatory care due to being homebound as a result of advanced frailty. Individuals accessing this service were already receiving HC for regular assistance with personal care. Referral to the home-based primary care program was through the usual family physician, and a requirement for admission was that patients agreed to leave their family physician and receive primary care from an HBPC team physician.

#### Home care (HC) cohort

In British Columbia, Canada, HC is initiated when a case manager working in the public home health system, uses a common set of criteria to assess an individual as requiring regular home support for personal care. Personal care includes the need for regular assistance with bathing, meal preparation, feeding, indoor ambulation and medication management. Referral to HC can be self-referral or more commonly by hospital staff or the family physician.

Once an individual is registered in the HC system, communication with the patient’s usual family physician occurs via fax and phone on an as-needed basis. There is no shared electronic medical record and/or routine conferencing of shared patients, and while some family physicians make emergency house calls, for the most part, patients are still expected to see their family doctor in their office. Prior to starting HC, the HC cohort had not yet received regular assistance with personal care.

A description of HBPC and HC services, eligibility and referral sources is provided in Table 1. The cohort already receiving HC, and starting HBPC as a result of becoming homebound due to advanced frailty, will henceforth be referred to as the HBPC group. The cohort just starting HC for regular assistance with personal care and still able to access usual primary care will henceforth be referred to as the HC group. Referral to both HBPC and HC is often triggered by an acute health crisis resulting in a decline in functional status.

**Table 1:** Comparison of home-based primary care (HBPC) and home care (HC) services, eligibility, and referral source in Vancouver, Canada, 2008-2013

Home-Based Primary Care (HBPC)	Home Care (HC)
Service description	
Longitudinal primary care by physician and nurse practitioners through regular house calls	“Usual” primary care, physician may or may not make house calls
Integrated team of registered nurses, and allied^a^ health professional working with family physicians and nurse practitioners	Home care nursing and allied^a^health professional services delivered through separate home health services program, communicating with family physician as needed
Regular team face to face meetings between physicians, nurse practitioners, and other team members, easy ad hoc communication amongst team members	No regular team meetings between physicians and HC team by fax or phone call
Team shares common electronic medical record across disciplines	Separate electronic record for physician and HC team with no interoperability
Dedicated 24/7 physician/nurse practitioner and capacity for responsive same-day/night care	Variation in 24/7 physician coverage, no capacity for responsive same-day/night care
Home support delivered by contracted out service through home and community care system	Home support delivered by contracted out service through home and community care system
Population Service Use Characteristics and Eligibility Criteria	
Already requiring and receiving regular assistance for personal care (HC services)	New onset of need for regular assistance for personal care (washing, meal preparation, feeding, medication management)
Unable to access usual primary care due to advanced frailty (homebound)	Able to access usual primary care
Referral source^b^	
Usual family physician, case manager at health unit of geographic catchment in which patient resides	Self-referral, physician, hospital

^a^Includes physiotherapy and occupational therapy

^b^Referral criteria have changed since completion of the study

#### Description of cohorts

**Fig. 1:**
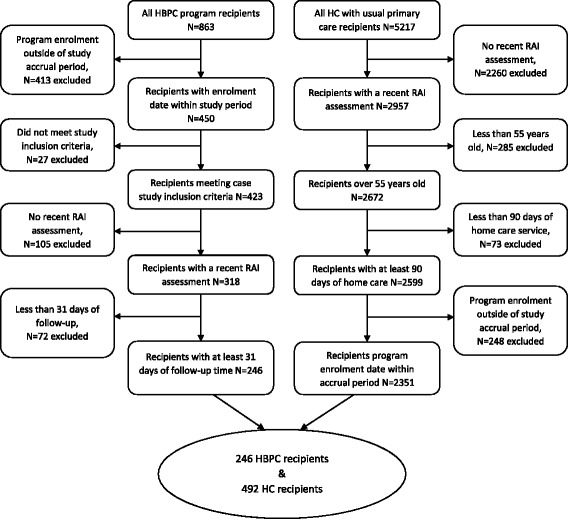
Attrition of home-based primary care (HBPC) and home care (HC) recipients included in study cohorts

This study was a before-after retrospective study of two different cohorts. It followed individuals accepted into the HBPC program between April 1, 2010 and June 30, 2013 (*N* = 863). Excluded from the HBPC cohort were those without a personal health number (PHN) (*N* = 7), individuals referred and not accepted into the program or those who declined service (*N* = 2), and those never seen by an HBPC physician or who had no follow-up (*N* = 18) (Fig. 1). Included in the study were recipients who had at least 31 follow-up days after enrollment in the program, and one or more Resident Assessment Instrument-Home Care (RAI-HC) [25] assessments completed between 270 days prior to or within 90 days after enrollment in the program.

RAI-HC are a series of standardized assessment tools for measuring cognitive and physical function [25]. Since 2007, all older adults receiving long-term home health services in the Vancouver health region (Vancouver Coastal Health) are formally assessed using this tool. Assessments are completed by long-term care case managers, entered into the HC electronic health record, and updated yearly or if there is a significant change in clinical status.

The second study population comprised individuals who had started to receive HC (case management and regular home support) over a similar study time period to the HBPC group, with the same inclusion/exclusion criteria (Fig. 1). Unlike the HBPC group, these individuals had not previously accessed HC, were not clients of HBPC, and were receiving usual primary care, through expected attendance at their family physician’s office.

The HC cohort was selected from 2351 individuals who had begun receipt of services after January 1, 2010 (had no observed service in the 21 months prior) and received a minimum of 90 continuous days of HC services before the end of the study, June 30, 2013. This cohort was selected by identifying a group with a similar distribution to the cohort receiving HBPC for the following characteristics: age (85+ versus < 85 years), sex, RAI-HC-derived CHESS [26] (levels 0-1 versus 2-5) and MAPLe [27] (levels 1-3 versus 4-5), and living situation (alone versus with others). CHESS, Hospital and Community Outcome Measures, is an algorithm derived from the MDS-RAI data and developed to detect frailty and instability in health. It identifies individuals at serious risk of decline. The scale ranges from 0 (no instability) to 5 (highest level of instability). [26] MAPLe, Method for Assigning Priority Levels, is also an algorithm derived from MDS-RAI data but is based on 14 indicators such as Activities of Daily Living (ADL) and cognitive functioning, falls and risk of institutionalization. It assigns a level from 1 (low) to 5 (very high) of functional dependency [27]. Due to the known association of mortality with hospital use, death after start of services was also included as an additional adjustment covariate. In order to adjust for secular effects, the study period for each population was divided into four distinct time periods, and the number starting HC services in each period was selected so it was also proportionate to the HBPC cohort. Twice as many individuals were included in the HC cohort compared to the HBPC cohort since the original pool of HC recipients was larger and allowed for this.

While the study attempted to construct two somewhat similar cohorts by selecting a subset of new HC recipients with RAI-HC characteristics that were similar to those of HBPC recipients, the groups were not assumed to be the “same” except for their status as two frail community-dwelling elderly cohorts starting two new services over the same time period. Both study populations were linked, through an anonymized unique identifier, to their records of hospital ED visit data and hospital discharge abstract data drawn from the Vancouver Coastal Health Region decision support databases. This technique has been previously described and validated [28]. Utilizing this data, cohorts’ ED visits were also described according to the Canadian Triage and Acuity Scale (CTAS). CTAS is a classification scale that groups patients into five levels of urgency when they present to the ED. It ranges from 1 (resuscitation) to 5 (non urgent) [29].

#### Descriptive data – Rate calculation

ED visit and hospital admission counts, and the number of days spent in hospital were tabulated for the periods before and after starting HBPC and HC respectively. The before period was from 21 months prior to the start of the respective program until the date of enrollment in the program. The after period started 31 days after HBPC or HC program enrollment until the study end for each patient. The study end was determined by admission to long-term care, death, or the end of the study (June 30, 2013), whichever came first. The rationale for the 31 day lag time in the after period was that it would give both programs a “grace” period to allow time for a program effect to develop. Days of observation, in the time periods before and after program start, were determined as the days when an individual was at risk of visiting the ED or being admitted to hospital. Days spent in hospital, admission to residential care, and death were censored from the days of observation denominator. Crude event rates were determined by dividing the count for the outcome of interest by the censored days of observation multiplied by 1000 patient days (PD).

#### Data analysis

All quantitative statistical analyses were conducted using SAS software, version 9.3 (SAS Institute Inc., Cary, NC, USA). Bivariate tests of comparison were completed to assess for the presence of significant differences between groups using Independent Samples t Test or Pearson’s chi-squared test for continuous and categorical variables respectively. Univariate Poisson regression modeling for each group was used to examine the association of HBPC and HC, and ED visit and hospital admission rates, by comparing adjusted rates of hospital use after versus before each service was started. Poisson regression analyses using the SAS PROC GENMOD procedure were utilized since overdispersed count data was used. Models were then adjusted for covariates that demonstrated a significant independent effect on the outcomes at a level of *p* < 0.10. The standard errors corrected for overdispersion were calculated by dividing the standard error by the square root of the scaled deviance. Corresponding 2-sided *p*-values were estimated according to the new standard error values.

The incidence rate ratios (IRR) and confidence intervals (CI), corrected for overdispersion, were determined for both the unadjusted and adjusted models by exponentiating both the parameter estimates and the parameter estimates +/− 1.96 times the corrected standard error. Separate models were run for each group because, while attempts were made to create some degree of similarity between groups using RAI-HC characteristics, it was anticipated that differences would remain in both measured and unmeasured variables. Separate models were run to allow for an understanding of these differences without the complication of a large number of difficult-to-interpret interaction variables. Since twice as many HC recipients were analyzed, sensitivity analyses were also carried out by randomly selecting half the sample size and re-running the regression models to ensure that none of the findings were an artifact of difference in cohort study size.

Ethics approval was obtained from the University of British Columbia’s Behavioural Research Ethics Board and the relevant ethics review boards within the Vancouver Coastal Health Authority region. These approvals included all administrative permissions necessary to access and use the study administrative data.

## Results

There were 246 individuals in the HBPC group and 492 in the HC group. Both groups had a similar distribution of sex, CHESS and MAPLe scores, and living alone. One third of the HBPC and one quarter of the HC group were over 90 years of age (33.3% versus 25.4%, *p* = 0.024) (Table 2).

**Table 2:** Baseline demographics and hospital use characteristics of home-based primary care (HBPC) and home care (HC) recipients

	HBPC Recipients *n* = 246	HC Recipients *n* = 492	*p*-value*
Mean age in years at admission (SD)	85.2 (9.2)	84.1 (9.1)	0.127
Minimum – maximum	55.8 – 103.9	56.5 – 103.1	
Age above 90 years, n (%)	82 (33.3)	125 (25.4)	***0.024***
Male, n (%)	87 (35.4)	175 (35.6)	0.957
CHESS Score^a^, n (%)			
0	47 (19.1)	94 (19.1)	1.000
1	79 (32.1)	158 (32.1)	
2	73 (29.7)	160 (32.5)	
3	35 (14.2)	61 (12.4)	
4	11 (4.5)	19 (3.9)	
5	1 (0.4)	0	
MAPLe Score^a^, n (%)			
1	4 (1.6)	12 (2.4)	1.000
2	13 (5.3)	40 (8.1)	
3	64 (26.0)	110 (22.4)	
4	107 (43.5)	237 (48.2)	
5	58 (23.6)	93 (18.9)	
Lives alone	132 (53.9)	266 (54.1)	0.962
Missing, n	1		
ED visit rate§ per 1000 PD (95% CI)	4.1 (3.8, 4.4)	3.0 (2.8, 3.2)	***<.0001***
CTAS^a^, n (%)			
1-3	451 (72.6)	613 (66.3)	***0.008***
4-5	170 (27.4)	312 (33.7)	
Hospital admission rate§ per 1000 PD (95% CI)	2.3 (2.1, 2.5)	1.3 (1.2, 1.4)	***<.0001***
Days spent in hospital§ per 1000 PD (95% CI)	41.8 (40.7, 42.8)	18.6 (18.2, 19.1)	***<.0001***

*SD* standard deviation, *CHESS* Hospital and Community Outcome Measures (An algorithm derived from the MDS-RAI data and developed to detect frailty and instability in health; identifies individuals at serious risk of decline; scale ranges from 0 (no instability) to 5 (highest level of instability)), *MAPLe* Method for Assigning Priority Levels (An algorithm derived from the MDS-RAI data and based on 14 indicators such as Activities of Daily Living (ADL) and cognitive functioning, falls, and risk of institutionalization; assigns a level from 1 (low) to 5 (very high) of functional dependency), *ED* Emergency Department, *PD* patient days, *CI* confidence interval, *CTAS* Canadian Triage and Acuity Scale (A classification scale that groups patients into five levels of urgency when they present to the ED, ranges from 1 (resuscitation) to 5 (non urgent))

*Tests of comparison included two independent samples t-test or Chi-square test; significant results are presented in boldface and italics

^a^Tests of comparison for CHESS Score, MAPLe Score, and CTAS carried out using binary variables: CHESS 0-1 versus CHESS 2-5; MAPLe 1-3 versus MAPLe 4-5; CTAS 1-3 versus CTAS 4-5

§*p*-value generated from univariate Poisson regression models; significant results are presented in boldface and italics

In the period before the start of HBPC, baseline ED visit and hospital admission rates (95% Confidence Interval (CI)) were 4.1 (3.8, 4.4) per 1000 PD and 2.3 (2.1, 2.5) per 1000 PD respectively. In the period before the start of HC, baseline ED visit and hospital admission rates (95% CI) for this cohort were 3.0 (2.8, 3.2) per 1000 PD and 1.3 (1.2, 1.4) per 1000 PD respectively (Table 2). Differences in baseline ED visit and hospital admission rates between groups were statistically significant (*p* < .0001) (Table 2). At baseline, HBPC recipients also presented to the ED with significantly higher acuity compared to HC recipients (as measured by a higher proportion of CTAS 1 to 3 at the time of presentation to the ED (*p* = 0.008)) (Table 2) and spent a greater number of days in hospital per 1000 PD (95% CI), 41.8 (40.7, 42.8) versus 18.6 (18.2, 19.1) (*p* < .0001) (Table 2).

In the period after receipt of HBPC, there was a non-significant decrease in ED visit rates (95% CI) from 4.1 (3.8, 4.4) visits per 1000 PD to 3.7 (3.3, 4.1) visits per 1000 PD (*p* = 0.332) and hospital admission rates (95% CI) from 2.3 (2.1, 2.5) admissions per 1000 PD to 2.2 (1.9, 2.5) admissions per 1000 PD (*p* = 0.726) (Table 3). Rates of days spent in hospital (95% CI) also had a non-significant decrease from 41.8 (40.7, 42.8) days per 1000 PD to 39.5 (38.1, 40.8) days per 1000 PD (*p* = 0.719) in the period after HBPC program enrollment (Table 3). Adjusted IRRs and 95% CI post receipt of HBPC were 0.91 (0.72, 1.15) for ED visit rates and 0.99 (0.76, 1.27) for hospital admissions (Table 4).

**Table 3:**
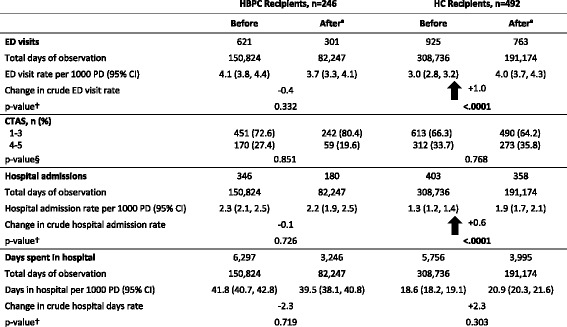
Crude rates of hospital utilization before and after starting home-based primary care (HBPC) or home care (HC)

*ED* emergency department, *PD* patient days, *CI* confidence interval, *CTAS* Canadian Triage and Acuity Scale (A classification scale that groups patients into five levels of urgency when they present to the ED, ranges from 1 (resuscitation) to 5 (non urgent))

^a^After enrolment in respective program including 30 day lag period

†*p*-value generated from univariate Poisson regression models; significant results are presented in boldface and italics

§*p*-value generated using Chi-square test for binary variable CTAS 1-3 versus CTAS 4-5; significant results are presented in boldface and italics

**Table 4:** Poisson regression, incidence rate ratios (IRRs) for emergency department (ED) visit and hospital admission rates by recipient type

	HBPC Recipients		HC Recipients	
	Unadjusted IRR (95% CI)	Adjusted^a^ IRR (95% CI)	Unadjusted IRR (95% CI)	Adjusted^a^ IRR (95% CI)
ED Visits				
After period versus before	0.89 (0.70, 1.13)	0.91 (0.72, 1.15)	***1.33 (1.14, 1.56)***	***1.34 (1.15, 1.56)***
Age < 80 (reference)				
Age 80-90	***0.72 (0.55, 0.93)***	***0.73 (0.56, 0.94)***	***0.65 (0.54, 0.78)***	***0.65 (0.55, 0.77)***
Age 90+	***0.58 (0.44, 0.78)***	***0.62 (0.47, 0.83)***	***0.59 (0.48, 0.73)***	***0.61 (0.49, 0.76)***
Male	***1.54 (1.24, 1.93)***	***1.45 (1.16, 1.80)***	***1.23 (1.05, 1.44)***	***1.14 (0.98, 1.34)***
Higher CHESS Score	***1.44 (1.15, 1.80)***	***1.43 (1.15, 1.78)***	***1.35 (1.15, 1.58)***	***1.32 (1.13, 1.54)***
Death	***1.34 (1.04, 1.73)***		***1.37 (1.06, 1.76)***	***1.40 (1.08, 1.80)***
Higher MAPLe Score	1.05 (0.83, 1.32)		1.09 (0.92, 1.28)	
Living alone	1.15 (0.92, 1.44)		0.93 (0.79, 1.09)	
Hospital Admissions				
After period versus before	0.95 (0.73, 1.24)	0.99 (0.76, 1.27)	***1.43 (1.20, 1.72)***	***1.46 (1.22, 1.74)***
Age < 80 (reference)				
Age 80-90	0.82 (0.61, 1.11)	0.84 (0.63, 1.13)	0.82 (0.66, 1.01)	0.82 (0.66, 1.01)
Age 90+	***0.70 (0.50, 0.98)***	0.80 (0.58, 1.10)	***0.68 (0.52, 0.89)***	***0.70 (0.54, 0.90)***
Male	***1.57 (1.22, 2.01)***	***1.45 (1.13, 1.86)***	***1.44 (1.20, 1.73)***	***1.31 (1.09, 1.57)***
Higher CHESS Score	***1.55 (1.21, 2.00)***	***1.52 (1.19, 1.95)***	***1.24 (1.03, 1.49)***	***1.21 (1.01, 1.44)***
Death	***1.71 (1.31, 2.24)***	***1.59 (1.22, 2.08)***	***1.99 (1.54, 2.56)***	***1.94 (1.50, 2.50)***
Higher MAPLe Score	1.14 (0.87, 1.49)		0.92 (0.76, 1.12)	
Living alone	1.15 (0.89, 1.47)		1.02 (0.85, 1.22)	

*HBPC* home-based primary care, *HC* home care, *CI* Confidence Interval, *CHESS* Hospital and Community Outcome Measures (An algorithm derived from the MDS-RAI data and developed to detect frailty and instability in health; identifies individuals at serious risk of decline; scale ranges from 0 (no instability) to 5 (highest level of instability)), *MAPLe* Method for Assigning Priority Levels (An algorithm derived from the MDS-RAI data and based on 14 indicators such as Activities of Daily Living (ADL) and cognitive functioning, falls, and risk of institutionalization; assigns a level from 1 (low) to 5 (very high) of functional dependency); significant results are presented in boldface and italics

^a^Adjusted for age, male, higher CHESS score; higher MAPLe score and living alone variables were non-significant and excluded from the adjusted models; standard errors corrected for overdispersion

In the period after receipt of HC, ED visit rates (95% CI) for this group increased significantly from 3.0 (2.8, 3.2) visits per 1000 PD before to 4.0 (3.7, 4.3) visits per 1000 PD after (*p* < .0001) and hospital admission rates (95% CI) increased from 1.3 (1.2, 1.4) admissions per 1000 PD to 1.9 (1.7, 2.1) admissions per 1000 PD (*p* < .0001). Post receipt of HC, the rate for days spent in hospital increased from 18.6 (18.2, 19.1) days per 1000 PD to 20.9 (20.3, 21.6) days per 1000 PD (*p* = 0.303) (Table 3). Adjusted IRRs (95% CI) in the period post receipt of HC were 1.34 (1.15, 1.56**)** for ED visit rates and 1.46 (1.22, 1.74) for hospital admission rates (Table 4).

In the adjusted models, male sex, higher CHESS score and death were almost all positively associated with ED visits and hospital admission rates for both groups (Table 3). Older age was inversely associated with ED visit rates in both groups and hospital admission rates for HC recipients only (Table 4). There were no differences in our results when we conducted sensitivity analyses to explore a potential effect due to the larger size that comprised the HC cohort.

At study end, a similar proportion from each group was admitted to a nursing home 0.22 (95% CI 0.17, 0.28) of HBPC (*N* = 54) and 0.24 (95% CI 0.21, 0.28) of HC recipients (*N* = 119), *p* = 0.481, data not shown), and a higher proportion of HBPC recipients died (0.21 (95% CI 0.16, 0.26), *N* = 51) compared to HC recipients (0.09 (95% CI 0.06, 0.12), *N* = 43, *p* = <.0001, data not shown). Among those who died, a higher proportion of HBPC recipients (0.45 (95% CI 0.31, 0.60), *N* = 23) versus HC recipients (0.37 (95% CI 0.23, 0.53), *N* = 16) died outside of hospital, although this result was non-significant (*p* = 0.439, data not shown).

## Discussion

This study used a retrospective before-after analysis of administrative data to assess the influence of two different services started in two different cohorts over the same time period. Both groups were community-dwelling seniors and both had experienced a change in health status over the same time period, prompting referral to either HBPC or HC. Both groups had a similar distribution of some case mix characteristics (sex, RAI-HC CHESS and MAPLe, and living situation) at the time of service start. Despite these similarities, those starting HBPC had a significantly higher rate of hospital services use compared to the HC group at baseline. The HBPC group was already receiving HC and was also therefore functionally frailer than the HC group at baseline. Based on the association of frailty and hospital use [30–32], the lower rates of hospital use seen at baseline by the HC versus HBPC group are, therefore, not surprising.

In the HBPC group, after start of HBPC, there was a non-significant decrease in ED visits and no change in hospital admission rates. How should the observed “no change” in hospital use between the time periods for the HBPC group be interpreted? If the expected trajectory of frail older adults’ hospital use is that of increased use over time, then the observed stabilization of rates seen in HBPC recipients after receipt of the service may be interpreted as a positive outcome. This interpretation is not unreasonable given the evidence for declining function and higher hospital use [30, 33]. The significantly greater proportion of HBPC recipients that died post service start compared to the HC group, and the known association of higher hospital use in the last 6 months of life [34], is another reason one might expect those receiving HBPC to have increasing rates of hospital use. However, despite considerable frailty and greater mortality of this group, hospital use rates remained unchanged.

For the HC group, in the period after starting HC services, there was a significant increase in hospital use for both outcomes such that the crude ED visit rate in the “after” period surpassed that of the HBPC group, and HC hospital admissions rates began to approximate those of the HBPC group. What factors might explain this? Firstly, these individuals were frail enough to begin to qualify for HC suggesting a major decline in health status. On this basis alone, the HC group would be expected to have increased hospital use over time. HC combined with usual primary care would be unlikely to bend this predictable rise given that HC services are only available during business hours, and usual primary care has variable provision of 24/7 crisis care with less than half of surveyed family physicians in Canada reporting an after-hours arrangement for their patients in 2015 [35]. Moreover, even when a health crisis occurs during daytime and business hours, neither HC nor usual primary care providers have the capacity to drop everything to attend to health crises in the home. In the absence of responsive care, the default action would therefore be to call the ambulance and go to hospital. Furthermore, communication between the “silos” of HC and primary medical care has been described as time-consuming and challenging [24], and only a minority of surveyed Canadian family physicians reported routine communication with HC and/or regular notification by HC about a change in their patients’ condition [35]. A future trial that randomizes receipt of HC versus HBPC to a previously HC naïve group of community-dwelling older adults, whose use is expected to rise over time, might provide valuable insights into the effect of HBPC on hospital use.

While limited, there has been some prior published US research on HBPC. Two randomized controlled trials found lower hospital costs ($17,805 USD versus $22,096 USD) [17] and lower ED visits and hospital admissions [13]. In both US studies, the study populations were younger and deliberately selected for socio-economic vulnerability, unlike the Vancouver HBPC group where there is no user fee for access and the services are covered by a single-payer public system. A third randomized controlled trial whose population was more similar to our HBPC population, found that team-managed HBPC for frail elderly US veterans resulted in a 22% relative decrease in hospital readmissions for a subset of individuals with severe disability only, and the effect was not sustained at 12 months [14]. A Quebec study demonstrated decreased functional decline and a smaller proportion of participants wishing to be institutionalized, but no influence on hospital use, among participants of an integrated home and primary care service delivery model, compared to controls [36]. One other Canadian before-after study showed a significant reduction in hospital use [15] with HBPC. However, the calculated rates in this study were not done with any offset of person-days of observation in the denominator making comparison difficult.

The research on HC and hospital use has also been mixed. Two US studies on HC intervention delivered by an advance practice nurse, and targeted at older adults recently admitted to hospital with congestive heart failure [37] and COPD [38] respectively, both found fewer readmissions in the intervention group. A Canadian randomized controlled trial of preventive home visits by a nurse to frail elderly clients also found no effects on mortality, institutional placement, or the combined use of health services utilization [39]. More recently a large Canadian report on the cost-effectiveness of HC found that hospital costs accounted for 30 to 60% of the overall costs for HC clients [40]. A major challenge of research evaluating both HC and HBPC models is the considerable heterogeneity in the services associated with each of these and the target populations for whom such services are delivered. Moreover, since there is a community expectation of the right to HC services for those who qualify in every province, randomization of access to usual HC would be unethical. However, a future “real world” trial with randomization to HBPC versus HC services for those starting to qualify for HC, may help to understand whether HBPC is able to influence hospital use at an earlier stage in the frailty trajectory. A challenge with such a trial would be the requirement that individuals leave their usual family physician, with whom many have had a longstanding relationship, when they are not yet homebound.

Besides a higher use of hospital services at baseline, one in five in the HBPC group died over the course of the observation period compared to one in eleven in the group of HC recipients (*p* < .0001). There are a number of possible reasons for this. Firstly, one third of the HBPC group was over 90 years of age compared to one quarter of the HC group. Those in the HBPC group are therefore more likely to die simply by virtue of being older. Second, despite similarities in distribution of RAI-HC measures, it is clear that the HBPC group was already substantially frailer than the HC group at baseline. Unlike the HC group that was home care naïve, those starting HBPC were already using HC services suggesting a greater baseline functional dependency. Also, unlike the HC group that continued to go to their family doctor’s office, individuals starting HBPC were frail to the point of being homebound and no longer able to access ambulatory primary care. Moreover, the HBPC group, at baseline, already had a significantly higher rate of hospital use compared to the HC group, suggesting higher morbidity in this group to begin with. Given the advanced age, greater frailty and higher morbidity at baseline, all of which are significant independent predictors of mortality, the fact that a higher proportion of HBPC recipients died over the study time period is not surprising.

It is also possible that the HBPC cohort were comprised of individuals who disproportionately chose to remain at home when acutely ill, foregoing potential access to life-extending services and opting instead for a home death. This is supported by the greater Canadian Triage and Acuity Scale (CTAS) levels (1, 2 and 3) at time of presentation to the ED seen in the HBPC compared to the HC group, both before and after the start of the HBPC service.

The presence of 24/7 responsive HBPC clinicians who provide pro-active advance care planning and symptom management for palliation at the time of a health crisis may further contribute to a greater proportion of families and patients opting for comfort care versus life extension. Forty-five percent of the HBPC compared to 3% of the HC group died outside the hospital. Although this difference was not statistically significant, possibly due to the small sample size for this outcome, it supports the hypothesis that more HBPC recipients may be opting for death at home over hospital and life prolongation. Research to explore the advance care planning decision-making of frail homebound recipients of HBPC for a “do not hospitalize” directive compared to other frail populations would be another important line of future research.

The study crude ED visit rates for both the HBPC and HC groups are higher than those reported for Vancouver nursing home care and assisted living residents (1.8 per 1000 PD and 3.4 per 1000 PD, respectively) [41] and for Ontario nursing home residents (2.1 per 1000 PD) [42]. This likely reflects the advanced frailty of homebound seniors who continue to live in the community with relatively lower levels of support compared to those in assisted living and nursing home settings. Indeed prior research from British Columbia has demonstrated a stabilizing effect of assisted living with 18,000 more hospital days in the year before compared to the year after moving to assisted living [43].

The association of male sex with higher rates of ED visits [44] and hospital admissions [45, 46] has been described in previous studies. Likewise the strong association of death as an independent predictor of hospital use has been widely described in health services research [47, 48]. Higher age was inversely associated with hospital use and both groups demonstrated a gradient effect of progressively lower adjusted incidence rate ratios for ED visits and hospital admissions with each advancing age group compared to those less than 80 years. Prior research from Ontario has also found significantly decreased hospital use by nursing home residents 90 years and over compared to individuals between 70 and 79 years in the last six months of life [49].

This study had a number of limitations. Firstly, like all before-after studies, the findings are subject to possible regression to the mean and the influence of secular effects. While we tried to partially address this by choosing to examine two community-dwelling senior populations, each starting new (but different) home-based services over the same time period, the populations were sufficiently different to make them non-comparable as true controls thus limiting the generalizability of conclusions. Second, like all retrospective observational studies there is risk of unmeasured bias and confounding. Despite these limitations, the study adds to a relatively small amount of research on HBPC, HC and hospital use [50, 51]. Further research is needed given both the expected higher use of acute health services with advancing age and the need to find models that support the desire of many older adults to remain in their homes as long as possible.

## Conclusions

This retrospective cohort study explored the effect of two different programs in two frail populations over the same time period in relation to their impact on hospital use. After enrollment in integrated HBPC, ED visit rates and hospital admission rates stabilized. After starting HC with usual primary care, ED visit and hospital admission rates for these recipients continued to rise. Although both populations were substantially different, results suggest that expansion of HBPC to individuals at an earlier stage of their frailty trajectory may be an opportunity to “bend the curve” of increasing hospital use seen in the HC cohort, thereby improving care and reducing cost.

## Abbreviations

CHESS: Hospital and Community Outcome Measures - An algorithm derived from the MDS-RAI data and developed to detect frailty and instability in health; identifies individuals at serious risk of decline; scale ranges from 0 (no instability) to 5 (highest level of instability)
CI: Confidence interval
CTAS: Canadian Triage and Acuity Scale - A classification scale that groups patients into five levels of urgency when they present to the emergency department, ranges from 1 (resuscitation) to 5 (non urgent))
ED: Emergency department
EMR: Electronic medical record
HBPC: Home-based primary care
HC: Home care defined in this cohort as the case management and regular receipt of home support for assistance with bathing, and/or meal preparation, feeding, indoor ambulation, and medication management
IRR: Incidence rate ratio
MAPLe: Method for Assigning Priority Levels - An algorithm derived from the MDS-RAI data and based on 14 indicators such as Activities of Daily Living (ADL) and cognitive functioning, falls, and risk of institutionalization; assigns a level from 1 (low) to 5 (very high) of functional dependency
PD: Patient days
PHN: Personal health number
RAI-HC: Resident Assessment Instrument-Home Care
